# NK and T Cell Subtypes in the Endometrium of Patients with Recurrent Pregnancy Loss and Recurrent Implantation Failure: Implications for Pregnancy Success

**DOI:** 10.3390/jcm12175585

**Published:** 2023-08-27

**Authors:** Anne-Sophie Braun, Kilian Vomstein, Elisabeth Reiser, Susanne Tollinger, Christiana Kyvelidou, Katharina Feil, Bettina Toth

**Affiliations:** 1 Department of Gynecological Endocrinology and Reproductive Medicine, Medical University of Innsbruck, Anichstraße 35, 6020 Innsbruck, Austria; anne-sophie.braun@i-med.ac.at (A.-S.B.); thomas.kilian.vomstein@region.dk (K.V.); elisabeth.reiser@i-med.ac.at (E.R.); susanne.tollinger@i-med.ac.at (S.T.); christiana.kyvelidou@i-med.ac.at (C.K.); bettina.toth@i-med.ac.at (B.T.); 2 Department of Obstetrics and Gynecology, The Fertility Clinic, Copenhagen University Hospital, Hvidovre, Kettegård Allé 30, 2650 Hvidovre, Denmark; 3 Recurrent Pregnancy Loss Unit, Copenhagen University Hospital (Rigshospitalet and Hvidovre Hospital), 2100 Copenhagen, Denmark

**Keywords:** reproductive immunology, uterine NK cells, immunophenotype, endometrium, miscarriage

## Abstract

Background: RPL and RIF are challenges in reproductive medicine. The immune system plays a pivotal role in endometrial receptivity, successful implantation, and pregnancy complications. Immunological changes have been associated with RPL and RIF. Understanding immune dysregulation especially in NK and T cell subtypes may lead to better diagnostic concepts and treatments. From July 2019 to August 2020 patients with RPL and RIF underwent a standardized diagnostic procedure including endometrial biopsies. Immune cell analysis was performed using flow cytometry. Patients were contacted in March 2023 and interviewed concerning their pregnancy outcomes following diagnostics. Results: Out of 68 patients undergoing endometrial biopsies, 49 patients were finally included. Live birth rates were high with 72% in RPL and 86% in RIF. Immune cell analysis revealed that patients with RPL had more cytotoxic CD56^dim^CD16^high^ cells, while RIF patients had more CD56^+^ uNK cells. RPL patients with pregnancy complications showed increased NKT cell percentages. Conclusion: Our findings suggest specific immune changes in RPL and RIF patients, offering potential therapeutic targets. Tailored immunotherapy based on endometrial immunophenotyping might be an option, but further research is needed.

## 1. Introduction

Recurrent pregnancy loss (RPL) and recurrent implantation failure (RIF) represent significant challenges in reproductive medicine, affecting numerous couples worldwide. The World Health Organization (WHO) defines RPL as ≥ 3 miscarriages, whereas the American Society for Reproductive Medicine defines it as > 2 consecutive pregnancy losses [1,2,3,4]. RPL occurs in 1–5% of couples actively seeking to conceive [1,3,5]. RIF is most commonly defined as the failure to achieve a clinical pregnancy after the transfer of three or more good-quality embryos during assisted reproductive technology (ART) [6,7]. Despite advancements in ART and the understanding of various etiological factors, the underlying mechanisms responsible for these conditions remain elusive. Recent research has shed light on the pivotal role of the immune system in the maintenance of endometrial receptivity and successful implantation [8]. Consequently, investigating the immune cell profile within endometrial biopsies has emerged as a promising avenue for unraveling the intricate interplay between immunological factors and reproductive outcomes.

The endometrium undergoes a complex series of changes during the menstrual cycle in preparation for embryo implantation. This process requires precise coordination between the embryo and the endometrium [8]. Any disruption can lead to RPL or RIF. Immunological factors, including various immune cell populations and their intricate signaling pathways, are known to modulate endometrial receptivity and implantation [9]. In particular, B cells, natural killer (NK) cells, macrophages, dendritic cells, and T-lymphocytes participate in the establishment of immune tolerance or inflammation at the feto-maternal interface. NK cells are a type of cytotoxic lymphocyte that plays a crucial role in innate immunity. Based on the expression of surface markers CD16 and CD56, NK cells can be subdivided into two primary subsets: CD56^bright^CD16^dim^ and CD56^dim^CD16^bright^. The former are primarily involved in cytokine production and are less cytotoxic, often found in secondary lymphoid tissues, while the latter are highly cytotoxic and are predominant in the peripheral blood. In the endometrium, uterine NK (uNK) cells are abundant during the luteal phase and early pregnancy and are mainly CD56^bright^CD16^dim^. They are involved in regulating vascular remodeling, promoting tissue repair, and modulating the immune response. NK cells also participate in the establishment and maintenance of pregnancy by interacting with trophoblast cells [10,11,12].

Monocytes are a type of white blood cell that circulates in the bloodstream and can migrate into tissues, where they differentiate into macrophages or dendritic cells. In the endometrium, monocytes/macrophages contribute to tissue remodeling, immune surveillance, and wound healing. They also play a role in the early stages of pregnancy, assisting with implantation and trophoblast invasion [13,14].

T cells are a type of lymphocyte that plays a central role in adaptive immunity. In the endometrium, T cells are involved in local immune responses, particularly in regulating inflammation and immune tolerance. They can differentiate into different subsets, such as regulatory T cells (Tregs), which help maintain immune balance and prevent excessive immune activation [15].

NKT cells are a specialized subset of T cells that have properties of both innate and adaptive immune cells. In the endometrium, NKT cells contribute to the regulation of local immune responses and tissue homeostasis. They can produce cytokines and interact with other immune cells, thus influencing the overall immune environment [16].

Studies have reported changes in the number and functional characteristics of immune cells (e.g., NK cells) in the endometrium of women with RPL and RIF [17,18,19,20,21,22]. While NK cells are known to be increased in early pregnancy, an exaggerated increase or decrease in endometrial NK cell numbers seems to be associated with RPL and RIF [17,23,24,25]. In addition, when focusing on different NK cell subtypes, an increase in cytotoxic NK cells and a decrease in regulatory NK cells can be seen in RIF and RPL [26,27].

Understanding these immune dysregulations could provide valuable insights into potential diagnostic markers and help us to establish specific therapeutic targets.

Herein, we aim to explore the intricate relationship between immune cell profiles in endometrial biopsy in patients with RPL as well as RIF in correlation to their reproductive outcome. By analyzing an extensive set of endometrial immune cells (B cells, NK cells, NKT cells, monocytes, and T cell subtypes, as well as uNK cell subtypes), we aim to identify changes in patients with RPL and RIF to gain further knowledge on these conditions and understand the immunological underpinnings of RPL and RIF [17].

## 2. Materials and Methods

### 2.1. Study Population

Patients with RPL/RIF who underwent a diagnostic work-up at our clinic between July 2019 and August 2020 were recruited for this study. Diagnostics were performed in non-pregnant patients with RPL and RIF according to the DGGG/OEGGG/SGGG and ESHRE guideline recommendations. Obstetric and medical histories were obtained, including age, gravidity, parity, number of miscarriages, and number of embryo transfers. Patients with two or more miscarriages were included in the RPL group and patients with at least three transfers of good-quality embryos without achieving a clinical pregnancy (detected by vaginal ultrasound) were assigned to the RIF group. Non-pregnant patients with RPL and RIF were screened for possible risk factors including hormone dysregulation (e.g., corpus luteum insufficiency), immunological disorders (antinuclear antibodies > 1:160), antiphospholipid syndrome using the revised Sapporo criteria (anticardiolipin antibodies (IgG ≥ 10 U/mL, IgM ≥ 5 U/mL), anti-ß2-glycoprotein (IgG ≥ 10 U/mL, IgM ≥ 10 U/mL), or lupus anticoagulant detection additionally to medical history), and acquired or inherited thrombophilia (deficiency of protein C/S, factor XII, or antithrombin, mutations in the factor V or prothrombin gene). Blood samples were taken in the follicular (between day 2 and 5 after menstruation) and in the mid-luteal phase (between day 5 and 8 after luteinizing hormone surge).

**Figure 1:**
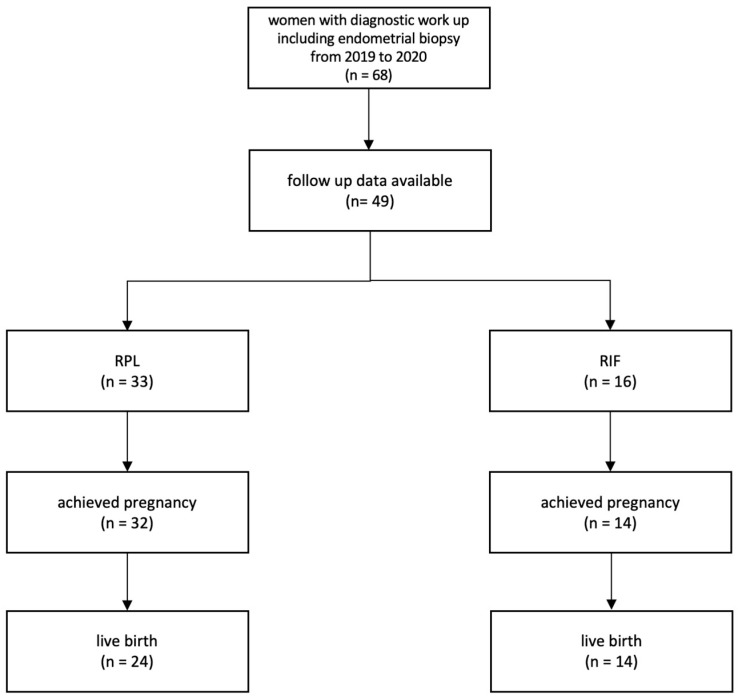
Study flow chart. recurrent pregnancy loss = RPL, recurrent implantation failure = RIF.

Endometrial biopsies were routinely performed using a Pipelle sampler (Pipelle^®^ CCD, Laboratoire CCD, Paris, France). Biopsies underwent immunohistochemical staining with CD138 (transmembrane heparin sulfate proteoglycan) to detect CD138-positive plasma cells. If more than 5 CD138-positive plasma cells/mm^2^ were identified, the patient was diagnosed with chronic endometritis. The biopsies were also stained immunohistochemically using CD56 as a marker for uterine NK cells. UNK cells were regarded as being elevated, when >300 cells/mm^2^ of the endometrial biopsy were found [21]. A summary of the study can bee senn in Figure 1. 

### 2.2. Therapeutic Strategies

All patients received treatment after the diagnostic process including the endometrial biopsy. All patients with RPL with idiopathic RPL or corpus luteum insufficiency received vaginal progesterone 2 × 100 mg 3 times daily during the luteal phase. This treatment was also administered in all patients with RIF after embryo transfer. Acetylsalicylic acid was prescribed in a dosage of 100 mg orally daily, starting with a positive pregnancy test until the first trimester screening and continuing the therapy depending on the risk for preeclampsia. LMWH was prescribed with a dosage of 40 mg (Lovenox^®^, Sanofi, Vienna, Austria) from a positive pregnancy test until a maximum of 6 weeks postpartum depending on the diagnosis and discretion of the treating physician. Patients with the diagnosis of a chronic endometritis received antibiotic treatment with 100 mg doxycycline for 14 days as first-line therapy. A second biopsy was performed to check for a successful treatment. If plasma cell numbers were still increased, a second-line antibiotic therapy with ciprofloxacin 1 g/day and metronidazole 1 g/day for 14 days was performed. Another biopsy after that confirmed treatment success in all cases. Patients with increased uNK cells received 20% Intralipid infusions (Fresenius Kabi Austria GmbH, B05BA02, 8 mL in 250 mL NaCL over 30 min) every 3 months before pregnancy and with onset of pregnancy every second week until the 12th week of pregnancy. Glucocorticoids were given at a dosage of 20 mg daily from positive pregnancy test until detection of fetal heartbeat.

To evaluate clinical outcomes, patients were contacted by phone between February and March 2023 and their further clinical history after the diagnostic work-up was recorded. Following verbal consent, the patients were asked about further pregnancies, miscarriages, live births, and treatment during the pregnancies. Pregnancy complications such as preeclampsia, gestational diabetes, and intrauterine growth retardation were recorded. Finally, n = 49 patients were included in the study.

### 2.3. Endometrial Biopsies

Endometrial biopsies were taken from n = 68 patients during the mid-luteal phase, defined as 7–10 days after luteinizing hormone (LH) surge. Tissue was collected in 5 mL Hank’s Balanced Salt Solution (HBSS, Sigma, H6648) supplemented with 2% FBS (Gibco, 10082147), washed in HBBS-FBS 2%, and cut. The tissue suspension was diluted in a final volume of 5 mL HBSS 2% FCS and digested with 0.25 mg/mL Collagenase D (Roche, 11088858001) and 0.12 mg/mL DNase I (Roche, 1010459001) at 37 °C for 30 min. At the end of the digestion, 20 mM EDTA (Invitrogen, AM9260G) was added, and the suspension was passed through a 100 μm and subsequently through a 30 μm cell strainer.

Cells were counted in a Neubauer chamber and washed in Dulbecco’s Phosphate Buffered Saline (DPBS)-FBS 1% (Lonza, 17-512F).

### 2.4. Immune Cell Analysis

**Figure 2:**
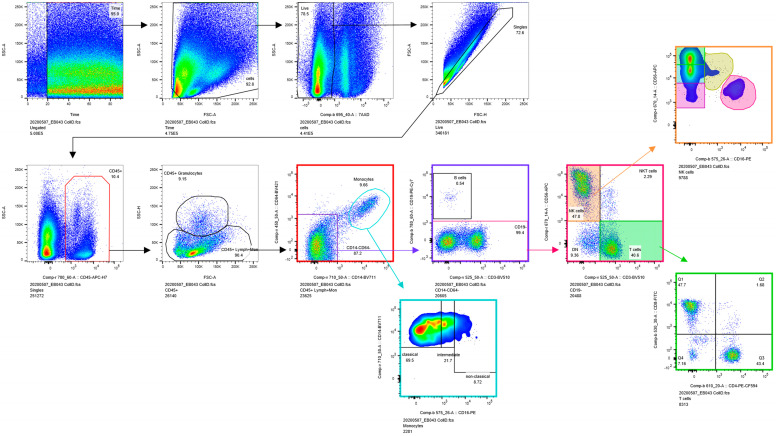
Gating Strategy.

Non-specific antibody binding was blocked with 1% Fc Blocking Reagent (Miltenyi, 130-059-901). For the staining, cells were incubated with fluorophore-labelled antibodies for 15 min at 37 °C (antibody list provided in Appendix A). 7AAD was added to a final dilution of 1:100 to exclude dead cells. Samples were analyzed in a BD LSRFortessa (BD Biosciences, San Jose, CA, USA) FC, where ≥ 5 × 10^5^ events were acquired for each sample. Flowcytometry data were analyzed with FlowJo (version 10.6.2 for Windows FlowJo Software, Becton, Dickinson and Company, Franklin Lakes, NJ, USA). Gating strategy is shown in the supplement (Figure 2).

### 2.5. Statistics

Further statistical analysis was performed with GraphPad Prism version 9.5.1 for Mac (GraphPad Software, La Jolla, CA, USA). In case of normally distributed raw data (as tested by the Shapiro–Wilk test with a significance level (alpha) of 0.05 and confirmed by a normal quantile–quantile plot), paired Student’s *t*-test was used. Not normally distributed data were compared using the Wilcoxon–Mann–Whitney test (*p* < 0.05 significant). Results are expressed as mean with standard deviation, median ± interquartile range, unless stated otherwise.

### 2.6. Ethical Approval

The study was conducted according to the guidelines of the Declaration of Helsinki and approved by the Ethics Committee of the Medical University of Innsbruck, Austria (EK Nr: 1210/2017, 15.02.2018). Signed informed consent was obtained from all subjects involved in the study.

## 3. Results

### 3.1. Study Population

In total, n = 68 patients underwent an endometrial biopsy between July 2019 and August 2020. These patients were contacted in March 2023 to gain further details on their pregnancy outcome. Finally, n = 49 patients were included in this study. Other than being unavailable by phone (n = 11), some patients (n = 8) also stopped trying to achieve pregnancy.

Demographic data are displayed in Table 1. Patients with RPL had experienced three (2.0–7.0) pregnancy losses, whereas patients with RIF had a median of three (3.0–4.0) embryo transfers (median number and IQR). Of the patients with RPL, n = 11 had a history of live births before the pregnancy losses. Patients with RPL had double the number (n = 3) of verified pregnancies in comparison to RIF patients (n = 1.5). The RPL group suffered triple the number of pregnancy losses (n = 3) compared to RIF (n = 1), whereas RIF patients showed higher numbers of embryo transfers (n = 3 vs. n = 0).

### 3.2. Clinical Data

Table 2 summarizes the diagnostic characteristics of RPL and RIF patients. There were no significant differences between the groups. However, the odds ratio suggests that RPL patients were more likely to have thrombophilia, while RIF patients tended to present elevated uNK cells. A total of n = 11 RPL/RIF patients suffered from at least one hereditary or acquired thrombophilia, whereas only n = 3 RPL/RIF patients were diagnosed with an immunological disorder (antiphospholipid syndrome as defined by the revised Sapporo criteria or twice positive antinuclear antibodies >1:160). As for the analysis of the endometrium, n = 6 RPL/RIF patients suffered from chronic endometritis and n = 14 RPL/RIF patients had elevated uNK cells.

Patients underwent the following treatments (Table 3): progesterone (83.7%), antithrombotic therapy (low molecular weight heparin (LMWH) (71.4%) and/or acetylsalicylic acid (34.7%)), immunomodulating therapy (corticosteroids (18.4%) and/or lipid infusions (26.5%)), antibiotic treatment (10.2%), or none (8.2%). A significantly higher number of patients with RIF (100%) received progesterone treatment when compared to patients with RPL (75.8%).

After a median time of 6.1 months following extensive diagnostics, n = 46 pregnancies were achieved resulting in n = 38 live births. The mode of conception was spontaneous in n = 23 (50%) and ART in n = 23 (50%), respectively. The mode of delivery was spontaneous in n = 19 (50%) and caesarean section in n = 19 (50%).

Of the n = 49 patients with RPL or RIF, 93.9% were able to achieve a clinical pregnancy (confirmed by the detection of a gestational sac via ultrasound). Livebirth rates were equally high in both groups: 72% and 86% respectively had a live birth. Over half of the RPL patients (58.3%) and one third of the RIF patients (35.7%) suffered from pregnancy complications, including preeclampsia, gestational diabetes, and intrauterine growth retardation. Results are demonstrated in Table 4. 

### 3.3. Endometrial Immune Cell Analysis

The general immune cell panel included CD45^+^ B cells, T cells, uterine NK cells, NK T cells, and monocytes. The T cell subgroup included CD4^+^ and CD8^+^ T cells. Five different subpopulations of NK cells were identified depending on the expression levels of CD56 and CD16 which were represented as follows: CD56^+^CD16^−^, CD56^+^CD16^low^, CD56^bright^CD16^−^, CD56^dim^CD16^−^ and CD56^dim^CD16^high^.

A comparison between the RPL and RIF group is displayed in Table 5. In the RIF group, a significantly higher percentage of cytotoxic NK cells (49% of CD45^+^) was present (RPL group: 37% of CD45^+^). The subgroup analysis showed that the percentage of CD56^dim^CD16^high^ NK cells was twice as high in RPL (6.5%) as in RIF patients (3.2%).

In addition, the immune characteristics of patients with RPL were compared to RIF (Table 6). A significantly higher percentage of NK cells was present among the RIF group (49.4% of CD45^+^) when compared to RPL (35.9% of CD45^+^). Patients with RPL had double the percentage of CD56^dim^CD16^high^ NK cells (6.7% of NK cells) compared to those with RIF (3.2% of NK cells).

When comparing the immune profiles of patients with RPL who did or did not suffer from pregnancy complications, patients with complications showed twice as high a percentage of NKT cells as those without (4.8% vs. 2.1% of CD45^+^) (Table 7).

A comparison regarding complications in pregnancy among patients with RIF could not be performed due to small numbers (n = 4 RIF patients with complications).

## 4. Discussion

The immune system plays a crucial role in regulating the processes involved in successful implantation and pregnancy maintenance. In particular, uNK and T cells have been shown to play important roles in regulating the implantation process by promoting maternal tolerance. In this study, we examined the immune cell composition of the endometrium in patients with RPL and RIF in order to identify potential immune dysregulation.

Our study did not reveal significant differences in most immune cell subsets beside uNK cells. While patients with RIF had a higher number of CD56^+^ uNK cells, patients with RPL showed a higher level of cytotoxic CD56^dim^CD16 [28]. The separation of CD56^bright^ NK cells with cytokine production and CD56dim NK cells with cytotoxic activity has been predominant in the literature for a long time [26,27]. However, more recent studies suggest a more diverse classification of NK cells [11,29]; therefore, five different subpopulations of NK cells were identified depending on the expression levels of CD56 and CD16. Our results are in line with another study analyzing the endometrial immune system, showing elevated levels of CD56^+^ uNK cells [18]. However, the present study is unique in its examination of multiple subtypes of NK cells and the finding that a specific subset of NK cells (CD56^dim^CD16^high^) is more prevalent in women with RPL compared to those with RIF.

We also compared the immune profiles of RPL patients with and without pregnancy complications. The increased percentage of NKT cells in patients with RPL and pregnancy complications is an intriguing finding as NKT cells are a unique subset of T cells that possess both T cell and NK receptors [30]. NKT cells play an important role in immune regulation, inflammation, and tissue repair in various (patho-)physiological conditions. The increased percentage of NKT cells in patients with RPL with pregnancy complications may reflect the pathogenesis of these complications. Previous studies have suggested that NKT cells play a role in regulating feto-maternal immune tolerance during pregnancy [31,32]. They have also been shown to secrete cytokines, thereby promoting the expansion of regulatory and suppressing the activity of effector T cells [33]. Therefore, the increased percentage of NKT cells in patients with RPL with pregnancy complications could be a response to an altered feto-maternal immune interaction that requires NKT cell involvement. Further research is needed to clarify the potential role of NKT cells in pregnancy complications.

Our study has some limitations. First, the sample size was relatively small; as such, larger studies are needed to confirm our findings. Second, the lack of a control group. However, obtaining an endometrial biopsy is an invasive procedure and therefore ethically unacceptable in healthy women.

Broad immunotherapy with corticosteroids for RIF or RPL lacks strong evidence to support its routine use, mainly due to small and inconsistent study groups and patient selection [6,34,35]. Focused immunotherapy with intravenous intralipid or high doses of intravenous immunoglobulin (IVIG) in early pregnancy could, on the other hand, be a beneficial option for patient subgroups with poor reproductive history identified by endometrial evaluation, as demonstrated in two studies [36,37]. Another potential therapeutical approach could focus on the microbiome of patients with RPL and RIF, as a positive outcome could be seen in a patient who received a vaginal microbiota transplantation in a proof of concept case study [38]. Future research should concentrate on a subset of patients to see if specialized immunotherapy tailored to each patient’s endometrial immunophenotype might be helpful for the reproductive outcome.

Overall, our study provides insight into the immune dysregulation that may contribute to RPL and RIF. Our findings suggest that the immune cell composition of the endometrium may differ between these two groups and that targeting specific immune cell subtypes may be important in the development of new treatments.

## Figures and Tables

**Table 1:** Patients’ characteristics at baseline.

Demographics	RPL (n = 33)	RIF (n = 16)	*p*-Value
Age (years) ^1^	35.55 ± 4.47	35.88 ± 4.75	0.8136
Gravidity (n =) ^3^	3.00 (2.5–5.0)	1.50 (0.0–4.0)	<0.0001
Parity (n =) ^3^	0.00 (0.0–2.0)	0.00 (0.0–2.0)	0.7150
Pregnancy losses (n =) ^2^	3.00 (2.00–7.00)	1.00 (0.00–1.00)	<0.0001
Embryo transfers (n =) ^2^	0.00 (0.00–0.00)	3.00 (3.00–4.00)	<0.0001

Demographics of the study population at baseline: RPL (n = 33), RIF (n = 16). The table shows age, gravidity, parity, number of pregnancy losses and embryo transfers. *p*-value (<0.05 significant), ^1^ mean (SD), ^2^ median (IQR), ^3^ median (min-max), recurrent pregnancy loss = RPL, recurrent implantation failure = RIF.

**Table 2:** Patient diagnostics.

Diagnostics	RPL (n = 33)	RIF (n = 16)	*p*-Value	Odds Ratio (CI)
	N (%)	N (%)		
Thrombophilia	9 (27.3%)	2 (12.5%)	0.2884	3.000 (0.5859–15.01)
Immunological disorders	1 (3%)	2 (12.5%)	0.2453	0.2188 (0.01473–2.067)
Chronic endometritis	3 (9.09%)	3 (18.7%)	0.3002	0.4333 (0.09315–2.088)
Elevated uNK cells	9 (27.27%)	5 (31.25%)	>0.9999	2.260 (0.2283–2.902)

Diagnostics of the study population: RPL (n = 33), RIF (n = 16). The table shows frequency (n) and percentage (%) of patients in each group who were diagnosed with thrombophilia, immunological disorders, chronic endometritis and/or elevated uNK cells. *p*-value (<0.05 significant), recurrent pregnancy loss = RPL, recurrent implantation failure = RIF.

**Table 3:** Treatment in RPL/RIF.

Therapy	RPL (n = 33)	RIF (n = 16)	*p*-Value	Odds Ratio (CI)
	N (%)	N (%)		
Progesterone	25 (75.8%)	16 (100%)	0.041	0.000 (0.00–0.8976)
Anti-thrombotic therapy	22 (66.67%)	9 (56.3%)	0.5374	1.556 (0.4512–5.054)
Immunotherapy	12 (36.4%)	6 (37.50%)	>0.9999	0.9524 (0.2986–3.164)
Antibiotics	2 (6.1%)	3 (18.8%)	0.3133	0.2796 (0.04685–1.542)

Treatment of the study population: RPL (n = 33), RIF (n = 16).The table shows the frequency (n) and percentage (%) of patients in each group who received progesterone, anti-thrombotic therapy, immunotherapy, and antibiotics. *p*-value (<0.05 significant), recurrent pregnancy loss = RPL, recurrent implantation failure = RIF.

**Table 4:** Pregnancy outcomes in RPL/RIF.

Clinical pregnancy rate	N (%)	*p*-Value	Odds Ratio (CI)
RPL	32/33 (97%)		
		0.2453	4.571 (0.48–67.90)
RIF	14/16 (86%)		
**Live birth rate**	**N (%)**	***p*-Value**	**Odds Ratio (CI)**
RPL	24/33 (72%)		
		0.3002	0.3810 (0.075–2.010)
RIF	14/16 (86%)		
**Pregnancy complications**	**N (%)**	***p*-Value**	**Odds Ratio (CI)**
RPL	7/24 (29.8%)		
		>0.9999	1.029 (0.2756–3.765)
RIF	4/14 (28.6%)		

Pregnancy outcomes of the study population: RPL (n = 33), RIF (n = 16). Clinical pregnancy rate, live birth rate, and pregnancy complications are displayed. *p*-value (<0.05 significant), recurrent pregnancy loss = RPL, recurrent implantation failure = RIF.

**Table 5:** RPL and RIF immune cell types.

Main Immune Cell Types (%of CD45^+^)	RPL (N = 33)	RIF (N = 16)	*p*-Value
CD19^+^ B cells ^2^	1.19 (0.585–3.18)	1.21 (0.55–2.26)	0.6639
CD3^+^ T cells ^2^	35.80 (32.60–45.75)	33.70 (27.25–37.08)	0.1022
CD56^+^ NK cells ^2^	37.00 (22.15–47.00)	49 (44.60–54.70)	0.0053
CD3^+^ CD56^+^ NKT cells ^2^	3.02 (2.055–4.485)	3.060 (2.020–3.960)	>0.9999
Monocytes ^2^	5.510 (4.315–11.35)	4.930 (3.500–8.430)	0.2767
**CD3^+^-T cell subtypes (% of CD3^+^ T cells)**			
CD4^+^ T cells ^1^	42.22 ± 11.11	41.81 ± 9.945	0.9005
CD8^+^ T cells ^1^	51.55 ± 12.17	53.69 ± 10.44	0.5493
DN (CD4^−^CD8^−^) T cells ^2^	2.780 (1.875–3.875)	3.095 (2.010–3.788)	0.6846
**CD56^+^-NK cell subtypes (% of CD56^+^ NK cells)**			
CD56^+^CD16^− 1^	40.31 ± 12.69	43.41 ± 7.187	0.3687
CD56^+^CD16^low 2^	5.330 (3.130–6.975)	6.620 (6.620–9.478)	0.6843
CD56^bright^CD16^− 1^	38.22 ± 12.52	43.55 ± 8.888	0.1344
CD56^dim^CD16^− 2^	2.110 (1.340–3.970)	1.905 (0.9325–3.115)	0.3546
CD56^dim^CD16^high 2^	6.450 (5.020–13.40)	3.220 (1.778–4.585)	0.0005

Immune cell types of the study population: RPL (n = 33), RIF (n= 16). The table shows the main immune cell types (% of CD45^+^), CD3^+^-T cell subtypes (% of CD3^+^ T cells) and CD56^+^ NK cell subtypes (% of CD56^+^ NK cells) of patients in each group. *p*-value (<0.05 significant), ^1^ mean (SD), ^2^ median (IQR), recurrent pregnancy loss = RPL, recurrent implantation failure = RIF.

**Table 6:** Differences in endometrial immune profile in RPL and RIF patients with live births.

Main Immune Cell Types (% of CD45^+^)	RPL (n = 24) (Live Birth)	RIF (n = 14) (Live Birth)	*p*-Value
CD19^+^ B cells ^2^	1.57 (0.6175–3.300)	1.210 (0.5650–2.895)	0.7958
CD3^+^ T cells ^2^	37.75 (34.43–52.70)	33.70 (25.75–36.63)	0.0655
CD56^+^ NK cells	35.85 (22.28–45.95)	49.35 (43.73–55.77)	0.0084
CD3^+^ CD56^+^ NKT cells	2.690 (2.053–5.073)	3.060 (2.145–4.045)	0.6947
Monocytes	6.155 (4.448–10.78)	4.930 (3.630–8.130)	0.246
**CD3^+^ -T cell subtypes (% of CD3^+^T cells)**			
CD4^+^ T cells ^1^	40.72 ± 8.752	41.76 ± 10.65	0.7454
CD8^+^ T cells ^1^	53.83 ± 10.16	53.79 ± 11.21	0.9912
DN (CD4^−^CD8^−^) T cells ^2^	2.705 (1.863–3.973)	3.095 (1.960–3.938)	0.6702
**CD56^+^ -NK cell subtypes (% of CD56^+^ NK cells)**			
CD56^+^ CD16^− 1^	40.89 ± 13.55	43.26 ± 7.438	0.5501
CD56^+^ CD16^low 2^	3.71 (2.673–8.798)	6.620 (2.523–8.833)	0.6483
CD56^bright^CD16^− 1^	36.73 ± 12.28	43.43 ± 8.44	0.0798
CD56^dim^CD16^− 2^	2.38 (1.643–4.520)	2.115 (1.078–3.243)	0.2504
CD56^dim^CD16^high 2^	6.720 (5.238–13.45)	3.220 (1.325–4.995)	0.0003

Immune cell types of the study population with live births: RPL (n = 24), RIF (n = 16). The table shows the main immune cell types (% of CD45^+^), CD3^+^ -T cell subtypes (% of CD3^+^ T cells) and CD56^+^ NK cell subtypes (% of CD56^+^ NK cells) of patients in each group. *p*-value (< 0.05 significant), ^1^ mean (SD), ^2^ median (IQR), recurrent pregnancy loss = RPL, recurrent implantation failure = RIF.

**Table 7:** Correlation between pregnancy complications and endometrial immune profile in patients with RPL.

Main Immune Cell Types (% of CD45^+^)	RPL (N = 17) (Normal Pregnancy)	RPL (N = 7) (Pregnancy Complications)	*p*-Value
CD19^+^ B cells ^2^	2.185 (0.6975–3.805)	1.015 (0.5450–2.650)	0.3632
CD3^+^ T cells ^1^	39.83 ± 15.92	46.45 ± 13.12	0.2929
CD56^+^ NK cells ^1^	34.71 ± 19.81	33.47 ± 12.66	0.864
CD3 ^+^CD56^+^ NKT cells	2.095 (1.948–3.025)	4.785 (2.613–6.728)	0.0085
Monocytes	6.895 (4.223–10.53)	6.115 (4.793–12.68)	0.7627
**CD3^+^-T cell subtypes (% of CD3^+^ T cells)**			
CD4^+^ T cells ^1^	41.61 ± 7.900	39.48 ± 10.13	0.5688
CD8^+^ T cells ^1^	52.59 ± 10.24	55.55 ± 10.32	0.4942
DN (CD4^−^CD8^−^) T cells ^2^	2.54 (1.773–3.800)	2.915 (1.718–4.348)	0.8859
**CD56^+^-NK cell subtypes (% of CD56^+^ NK cells)**			
CD56^+^CD16^− 1^	39.44 ± 12.83	42.91 ± 14.95	0.5484
CD56^+^CD16^low 1^	5.081 ± 3.308	6.605 ± 5.035	0.3795
CD56^bright^CD16^− 1^	38.79 ± 11.70	33.84 ± 13.11	0.3412
CD56^dim^CD16^− 2^	2.32 (1.848–4.145)	2.46 (1.4–4.960)	0.8294
CD56^dim^CD16^high 2^	7.790 (5.757–13.35)	6.055 (5.030–17.70)	0.5458

Immune cell types of the study population with RPL pregnancy complications: normal pregnancy (n = 17), pregnancy complications (n = 7). The table shows the main immune cell types (% of CD45^+^), CD3^+^-T cell subtypes (% of CD3^+^ T cells) and CD56^+^ NK cell subtypes (% of CD56^+^ NK cells) of patients in each group. *p*-value (<0.05 significant), ^1^ mean (SD), ^2^ median (IQR), recurrent pregnancy loss = RPL, recurrent implantation failure = RIF.

## Data Availability

Not available due to privacy restrictions.

## Appendix A

**Table A1:** Fluorophore labelled antibodies used for the staining of endometrial cells.

Antibody	Clone	Company
CD3	HIT3a	BD
CD4	RPA-T4	BD
CD8	HIT8a	BD
CD14	MᴪP9	BD
CD16	3G8	BD
CD19	HIB19	BD
CD45	2D1	BD
CD56	AF12-7H3	Miltenyi
CD64	10.1	BD
